# Empowering the Research Community to Investigate Misconduct and Promote Research Integrity and Ethics: New Regulation in Scandinavia

**DOI:** 10.1007/s11948-022-00400-6

**Published:** 2022-11-17

**Authors:** Knut Jørgen Vie

**Affiliations:** TIK Centre for Technology, Innovation and Culture, University of Oslo, Sognsveien 77B, 3. Et., 0855 Oslo, Norway

**Keywords:** Research integrity, Research ethics, Codes of conduct, Scandinavia

## Abstract

Researchers sometimes engage in various forms of dishonesty and unethical behavior, which has led to regulatory efforts to ensure that they work according to acceptable standards. Such regulation is a difficult task, as research is a diverse and dynamic endeavor. Researchers can disagree about what counts as good and acceptable standards, and these standards are constantly developing. This paper presents and discusses recent changes in research integrity and ethics regulation in Norway, Denmark, and Sweden. Recognizing that research norms are developed through practice and are therefore unsuited for comprehensive national regulation, the Scandinavian countries focus on empowering the research community to regulate itself instead, except for the most severe cases of misconduct. This empowerment takes the form of giving research institutions tools and investigatory powers while also holding them responsible for ensuring that both the institution and individual researchers are up to date on relevant norms. In this way, the Scandinavian governments seek to avoid some of the challenges found in more legalistic approaches, which risk lagging behind the continuous development of research norms and can be insensitive to the fact that different disciplines have different norms. While the new approach in Scandinavian has several potential benefits, it also involves potential trade-offs and limitations. The new laws can create confusion about what researchers are allowed to do. Another issue is that it only addresses the fundamental drivers of misconduct to a limited extent.

Norway and Denmark revised their research ethics and integrity legislation in 2017, and Sweden did the same in 2019. All three countries have adopted a similar approach. On the one hand, they have identified some of the most egregious forms of misconduct and introduced procedures at the national level for handling such cases. On the other hand, they leave it to research institutions to define and handle less severe breaches of academic norms, and to ensure that research is conducted in a trustworthy manner. The laws try to empower institutions in order help them live up to this responsibility, by giving them certain privileges and tools. The Scandinavian governments have given institutions this combination of responsibilities and powers based on an explicit recognition that research integrity and ethics is too complex and dynamic to be regulated comprehensively through law.

In this paper, I present the arguments in the proposals (bills) of the legislation to the respective parliaments of the Scandinavian countries. These are currently only available in the Scandinavian languages and making these discussions available in English is an important contribution to the international debate on the regulation of research integrity and ethics. Using the bills as a source, I ask who do the laws hold responsible? For what? And how? In this way, I identify the actors each of the countries holds responsible for promoting integrity and ethics, what each of the countries takes these concepts to mean, and what they require of the actors in practice. Subsequently, I discuss the potential of the new laws to overcome the limitations of more rigid systems, while also highlighting some early signs that universities in Scandinavia are struggling to translate the laws into practice.

## Regulating Research Integrity and Research Ethics

Researchers are sometimes dishonest, and sometimes their work causes harm (Fanelli, 2009). The first is usually discussed in the academic literature under the heading *research integrity*, and the latter under the heading *research ethics*. Research ethics is primarily concerned with the potential negative effects research processes and findings can have. Examples include harming non-consenting research subjects or creating technologies that threaten life or health (Beauchamp & Childress, 2009). Research integrity is primarily concerned with the trustworthiness of research and the unwanted acts that can detract from it. The literature typically labels dishonest behavior in research as misconduct, fraud, or questionable research practices (QRP). Outright misconduct and fraud tend to be operationalized as three types of unwanted behavior: fabrication and falsification of data, and plagiarism (FFP) (Resnik et al., 2015). QRP is a more open category that includes acts that have the potential to detract from the trustworthiness of research without constitution outright fraud (Resnik, 2003).

Governments are important funders of research and are therefore interested in the ethical and trustworthy conduct of research. Both the regulation of research integrity and ethics have followed in the wake of historical events. Before these events, researchers were for the most part entrusted with the responsibility to ensure the integrity and ethical conduct of research through *self-regulation* and most governments took a *laissez faire* approach. As Robert Merton claimed in 1942, the mutual policing of researchers ensured that research was virtually free from fraud (Merton, 1973), and regulation was therefore considered unnecessary. Research integrity did not emerge as a serious concern until the 1970s, following a series of scandals involving dishonesty among researchers from the United States. Policymakers realized that researchers are not always trustworthy and decided to put research under increased regulation in order to ensure its integrity. This resulted in a congressional investigation and the establishment of the U.S. *Office of Research Integrity* (Resnik, 2003). Other countries followed suit, partially based on their own discoveries of misconduct. The regulation of research ethics has also resulted from historical experiences, such as the Second World War, which led to efforts for the protection of research subjects, such as the Declaration of Helsinki (World Medical Association, 2013).

Attempts at promoting integrity and ethics through regulation are, at times, highly contentious, and introduce new problems to the practice of research. An overview of such issues provides an important context for the present paper, as the new laws in Scandinavia must overcome them in order to achieve their goals, without at the same time becoming too burdensome. The American approach, with its *Institutional Review Boards* (IRB), is an example of a system that has received significant criticism. The purpose of the boards is to protect human research subjects through ethics review of research projects, an endeavor that typically falls within the category of research ethics. However, some also involve themselves in questions regarding research integrity (Klitzman, 2011). Redman and Caplan (2021) have recently argued that the handling of misconduct in research should be an integral part of the purpose of IRBs, as misconduct has the potential to harm research subjects.

Criticism of the IRB system has turned into something of a subgenre in the research ethics and integrity literature, at least until its recent revision. The American system has, among other things, hampered methodological innovation, blocked potentially controversial research to protect institutional reputations, and subjected social science to controls more suited for biomedical fields (Israel, 2015; Schrag, 2009, 2010). Some have even accused it of needlessly hindering research at the cost of human lives, for example by introducing a ban on studying resuscitation, since patients in such cases are unable to provide informed consent (Hiller et al., 2005). Researchers have also accused research ethics systems in general of more fundamental flaws, such as displacing the moral order that existed in research before its introduction, turning researchers from critically assessing ethical situations to ritualistically complying with the rules (Heimer, 2010). Research ethical rules can also give the impression that they are comprehensive, while no such document can cover all the complexities of moral life (Macfarlane, 2009). Such rules cannot take all the particularities of the situations and actors in question into account, and they are often silent when it comes to how they should be put into practice.

The regulation of research integrity and ethics is difficult because research is a dynamic endeavor. This dynamism make regulation difficult, as it will lag behind the most recent developments. What the research community accepts as appropriate research practices is constantly evolving. Randomized controlled trials were not introduced until the 1950s (Stolberg et al., 2004). Peer review in the form of written feedback from anonymous reviewers was not standard practice until the 1970s (Baldwin, 2018). Following the replication crisis, research is seeing an increased focus on openness, transparency, and control, in the form of open science and pre-registration of research protocols.

Another difficulty in regulating research integrity is the fact that different fields and disciplines have different standards (Lamont, 2009; Peels et al., 2019). This diversity can be a source of conflict if one attempts to create universal rules covering the conduct of research. For example, a salient issue among the social sciences is that concerns from biomedical fields dominate the regulation of research ethics (Israel, 2015). These concerns are not always a good fit for the social sciences. Furthermore, researchers from different countries differ in what they consider the most prominent issues in research integrity and ethics (Li & Cornelis, 2020). Establishing universal rules risks neglecting local ethical contexts and values.

A final challenge in regulating research integrity and ethics worth mentioning here is the fact that the literature has not reached a consensus regarding the content of these terms, and the relative seriousness of various forms of misconduct. This discussion is ongoing (Kuroki, 2018; Shaw, 2018), and it is therefore no surprise that regulation has been imperfect. We cannot expect regulators to be better at determining the norms of research than the researchers themselves.

In the literature, there is currently a discussion about whether certain forms of misconduct should be criminalized, particularly in the biomedical fields, and China has announced that they will implement an extensive system for punishing misconduct (Dal-Ré et al., 2020). There are significant difficulties in weighing the relative seriousness of various forms of misconduct (Bülow & Helgesson, 2019), and as shown above, formalizing the content of research integrity and ethics is challenging on account of its dynamic and diverse nature. Criminalizing misconduct, therefore, involves risks such as excluding novel forms of unwanted behavior from punishment or enforcing outdated methodologies that were best practice at the time of regulation. However, if the research community cannot ensure that research is conducted with integrity, governments can feel forced to turn to stricter measures, as the Chinese are now doing.

An alternative to both a laissez-faire approach and criminalizing misconduct is *meta-regulation* (Coglianese & Mendelson, 2010; Gilad, 2010). The approach aims at “exploiting the information advantages of those actors to be regulated by leveraging them into the task of regulating itself” (Dorbeck-Jung & Shelley-Egan 2013, p. 55). Rather than creating a list of acceptable and unacceptable behaviors, meta-regulation aims to ensure that the actors in question regulate themselves, by ensuring that they understand and take their responsibilities seriously, since these actors are better positioned to identify, interpret, and even develop the relevant norms. This approach is a form of enforced self-regulation, where measures are introduced to ensure that self-regulation takes place and is efficient. This can, for example, take the form of handing the relevant actors tools and powers to enforce their norms, introducing structures and arenas for self-regulation, and mandating that relevant norms are disseminated through training. The approach has been applied in ensuring that nanotechnology, a field with rapid development and uncertain outcomes, is conducted responsibly (ibid.).

In the case of research integrity and ethics, this approach constitutes a middle ground on a spectrum where a laissez faire/self-regulation approach and criminalization are the two extremes. The approach starts from a recognition that researchers should produce and enforce their own norms, while also recognizing that researchers and research organizations need structures and powers in order to take on this responsibility to a degree where misconduct and unethical research is adequately dealt with. As meta-regulation moves responsibility for regulating research closer to the practice of research, this approach has the potential to overcome some of the regulatory challenges related to the fact that research is a diverse and dynamic endeavor.

The discussion section in the present paper aims to highlight the extent to which the Scandinavian laws include and attempt to balance the three approaches to regulation mentioned above, namely a self-regulation/laissez faire approach, meta-regulation, and criminalization. This will contribute to answering the research question, as it provides a theoretical lens for understanding the nature of how the laws hold different actors responsible, and for understanding the nature of their responsibilities.

## The Context of the New Regulation in Scandinavia

The new Scandinavian laws were developed in the context of regulatory efforts internationally. This section will give a brief overview of this context. The SATORI-project^1^produced a report on European legal frameworks that govern research ethics, both on the European and national levels (Rangi & Warso, 2015). This report is relevant as it maps the European system right before the revision of the laws in the Scandinavian countries and thus captures the context of these revisions. Among other things, it maps the regulation of research integrity. The project shows that most of the documents that govern research in Europe adopt a *principled* or *rule-based* language. They list dos and don’ts, an approach on the criminalization side of the spectrum.

Comparisons of legal frameworks and codes regulating integrity is a complex matter. As the SATORI-project states, such regulation is found at several different levels and in various organizations, in addition to the legislative level. In their words, codes of conduct and policy documents are created by “… research integrity agencies, science-funding organizations, or by universities. Professional groups have their own codes of conduct partly devoted to research activities”, adding that “Principles of scientific integrity are rarely laid down in legally binding acts” (Rangi & Warso, 2015, p. 16). Research integrity regulations also differ in scope. Some approaches treat misconduct and ethical issues as separate matters, while others treat such issues in one and the same regulation.

In one of their reports, which also precedes the recent changes in Scandinavia, the PRINTEGER-project^2^ finds that “… only a limited number of European countries have adopted legislative instruments explicitly dealing with research integrity and scientific misconduct” (Fuster & Gutwirth, 2016, p. 5). They list Norway, Denmark, Finland, and Spain when discussing this approach. Even though the report shows that it is uncommon to formalize research ethics and integrity in law, local codes of conduct can still play a role in investigations of misconduct, and they are used in legal proceedings. When taking a job as a researcher, one often commits oneself to codes like, for example, the codes of conduct at one’s research institution, and breaking these codes can lead to consequences and litigation, even though they are not hard-law instruments, in the form of labor disputes (Freckelton, 2016). Therefore, the lack of research integrity and ethics regulation on the national level does not mean that law plays no role in dealing with such issues.

While the legislation in Scandinavia is now similar enough to make it meaningful to discuss the three laws as examples of the same type of approach, this convergence is recent. NordForsk, an organization that funds and coordinates research in the Nordic countries, arranged a seminar in 2014. They gathered experts to map the regulation of research ethics and integrity within their area of operation.^3^ The seminar resulted in a report (NordForsk, 2015) where they found significant differences in national guidelines and procedures, and they concluded by calling for increased cooperation. They argue that one of the merits of the existing systems was self-regulation, as opposed to juridification of research integrity, which has been a significant theme in the subsequent developments.

## Methodology and Analysis

While the laws and parliamentary propositions I want to discuss here have significant overlap, I will present the different countries' approaches separately to show the nuances between them and make their formulations of research integrity available internationally. I put particular focus on the parliamentary propositions. In the three countries, these are important legal sources that elaborate on the contents of the statutes and attribute responsibilities to actors not mentioned in the statutes themselves. I present them in the order they were enacted. Research ethics and integrity regulation is complex and multifaceted in all the countries included and goes beyond the three laws discussed here. For example, all three countries have separate laws regulating biomedical research and informed consent to comply with the Declaration of Helsinki. I have excluded these and other regulation that only cover a subset of research to focus on the most recent developments, which cover all research disciplines. All quotes and terms were translated into English by the author unless otherwise stated.

The analysis was conducted using NVivo. The approach follows one of Asdal and Reinertsen (2021) suggested approaches to document analysis, namely studying documents as tools of governing. This approach emphasizes the need to go beyond the content of documents, by including an analysis of what documents *do* or aim to do. In the present case this means going beyond capturing how the bills operationalize key terms, by also looking at how these documents try to hold certain actors accountable.

Each of the three parliamentary propositions were coded individually. The coding started from three initial deductive codes (Creswell, 2014). Firstly, the stated purpose of the law. This captures how the respective governments understand the problems regarding fraud and unethical research, which is foundational to how they operationalize key concepts and who they take the most important actors to be. Secondly, the acts that the law seeks to deter, along with the various terms and definitions used to describe misconduct, research ethics, and research integrity. This captures how the respective governments understand the norms they expect the research sector to live by. Thirdly, following Davies and Lindvig (2021), the responsibilities attributed by the law, along with their corresponding recipients. This allows for a discussion of the nature of the regulation of research integrity and ethics in the three countries, i.e., the extent to which researchers are expected to regulate themselves (laissez faire), criminalization, and meta-regulation. When a new actor was introduced in the proposition, a new node was created, where the actor’s responsibilities were coded. The results from the deductive coding, where all relevant statements were coded, were subsequently analyzed through an inductive and thematic approach (ibid.), in order to create the summary below of what the three countries take to be the main concerns and actors.

The presentation of the findings excludes most procedural duties and issues such as deadlines and the composition of committees to focus on the normative questions that are more relevant to the international debate.

## Findings

### Norway

#### Purpose

The Norwegian law is titled *Act on the organization of research ethics* (Prop 158 L (2015–2016)). It was presented to Parliament in 2016 and enacted in May 2017, and replaced and expanding on an earlier law from 2006. The proposition states that the law has two purposes. Firstly, it seeks to strengthen research ethics in Norway by formalizing the responsibilities of researchers and research institutions, and by creating a national system for promoting research ethics. Secondly, the introduction of the new law was motivated by a need to expand the previous law of 2006 and rectify some ambiguities in the old system, as the ministry believed that it led to too much local variation among the research institutions.

#### Key Terms and Scope

The main concern of the law is *recognized research ethical norms*. *Research misconduct* is defined as *severe* deviance from such norms, especially but not exclusively FFP. Misconduct is thus treated as a matter of research ethics. While the literature tends to distinguish between research ethics on the one hand and research integrity on the other, the Norwegian approach does not make such a distinction. This move is deliberate, and the ministry writes that they most of the time will translate the English term “research integrity” as “research ethics”. The law treats less severe breaches with recognized research ethical norms differently than outright misconduct, stating that “*The content* in research ethics, what counts as good research practice, good research habits, good research ethics, is still up to the research community to figure out” (p. 9). *Good research practice*, as used in this quote, is later equated with *recognized research ethical norms*. While some of the most severe forms of dishonesty and misconduct are defined in the law, less severe breaches are up to the research community to define and fill with content. The government recognizes that it is not in a good position to do this work as the norms of research result from the continuous self-regulation of research. However, the proposition adds that research ethics, to a significant extent, is defined in national and international codes of conduct and guidelines and expects the research community to develop and improve such documents continuously.

#### Responsibilities

The three most prevalent categories of actors held responsible by the law are researchers, research institutions, and research ethics committees. Additionally, the proposition briefly discusses the responsibilities of research funders, mentors and supervisors, and society in general. Under the law, both researchers and research institutions are responsible for ensuring that research is conducted in accordance with recognized research ethical norms. Researchers are responsible for keeping themselves informed about the relevant norms and acting according to these. Following the relevant norms is required throughout the entire research process, from planning to reporting. Additionally, researchers must familiarize themselves with relevant laws and international guidelines.

While research institutions are also responsible for ensuring that research is conducted in accordance with research ethical norms, their responsibility is not to control the research process directly but rather to ensure that certain systems and conditions are in place. According to the proposition, the responsibilities of institutions fall within two categories. The first is preventative and involves promoting research ethics in the day-to-day practice of research by making it an integral part of the institution’s culture through training, encouraging discussions about ethics, and being careful about introducing incentives that can promote unwanted behavior. The second involves uncovering and handling all forms of unwanted behavior. Research institutions must create codes of conduct, create procedures for handling unwanted practices, create a committee for handling such practices, and punish those who deviate from the norms. They must also handle all suspicions of unwanted behavior, even less severe cases. However, they can handle less severe cases in a cursory manner; without involving the committee.

In support of the research community, the law provides a mandate for several national research ethics committees, which builds on the previous law. It establishes a national investigatory committee tasked with investigating the most egregious breaches of the norms and supporting research institutions in their investigations. Additionally, three research ethics committees covering different academic fields are established on the national level. Their primary purpose is to advise researchers on specific ethical issues. They also create national ethical guidelines with input from the research community. Finally, the law establishes regional committees tasked with the evaluation and approval of medical research and a national committee that handles appeals of the decisions at the regional committees. This last set of committees primarily apply a separate law covering medical research and have been established to ensure compliance with the Declaration of Helsinki.

### Denmark

#### Purpose

The Danish law, titled the *Act regarding scientific dishonesty*, was presented for Parliament in January 2017, and enacted in July the same year (Lovforslag nr. L 117 (2016–2017)). The law's stated purpose is “to strengthen the trustworthiness and integrity of Danish research” (§1) by establishing procedures for handling cases of *scientific dishonesty* and *questionable research practices*. The law builds on an already established national committee for investigating scientific dishonesty, enacted through the Danish *Act regarding agencies in the research sector*.^4^

#### Key Terms and Scope

The law distinguishes between *scientific dishonesty* and *questionable research practices*. Scientific dishonesty is defined as “*Fabrication, falsification, and plagiarism committed willfully or gross negligent in planning, performing, or reporting of research*” (§3). The law defines questionable research practices as “*Breaches of current standards on responsible conduct of research, including those of the Danish code of conduct, and other applicable institutional, national and international practices and guidelines on research integrity*” (§3).^5^ While the first category is defined as specific acts, the latter is left to research institutions to fill with content through practice and by creating codes of conduct and guidelines. As the proposition refers to the national guidelines for research integrity in Denmark (Ministry of Higher Education & Science, 2014), along with other such documents, what questionable research practices entail is already codified to a significant extent.

#### Responsibilities

Since the scope of the Danish law is the handling of unwanted practices, the two most prevalent categories of actors to which the law attributes responsibilities are research institutions and the *Danish Committee on Research Misconduct*. The minister of research and education is given responsibility for oversight. Notably, the proposition does not go into the responsibilities of individual researchers beyond establishing categories of behavior that they must avoid. The proposition discusses the role of researchers in reporting misconduct but also states that this role can be filled by “anybody”. However, the proposition does hold the research community responsible for continuously developing what it means to do research responsibly, and researchers are responsible as part of this community.

Research institutions are handed a duty to report suspicions of scientific dishonesty to the committee on research misconduct and to gather and hand over the relevant facts regarding the case. On the other hand, questionable research practices are left to the research institutions to deal with themselves. This duty involves creating codes of conduct and guidelines for how questionable research practices are to be handled. Dealing with less severe cases of unwanted practices does not have to involve a formal process. For example, leaders can handle such cases by discussing the problem with the researcher in question. More severe cases, that nonetheless fall outside the definition of scientific dishonesty, can be punished at the discretion of the research institutions. Research institutions can establish committees for investigating such cases or establish committees in collaboration with other research institutions.

The responsibility of the committee on research misconduct is to investigate accusations of academic dishonesty, to ensure the equal treatment of such accusations. Research institutions and individual researchers can report their suspicions to the committee. Researchers can report themselves if they feel that this is necessary to clear their names. The committee can take on cases at its discretion, but the proposition states that it should do so only under exceptional circumstances. The committee can reject cases if they fall outside of its jurisdiction, if they are groundless, or if the costs of handling a case are not in proportion with the severity of the case. The committee can also send the case back to the research institutions in question if they find that it is not severe enough to rise to the level of scientific dishonesty but still meet the criteria for questionable research practices.

### Sweden

#### Purpose

The Swedish law is titled *Act on responsibility for good research practice and examination of research misconduct* (Prop., 2018/19:58).^6^ It was presented to Parliament in February 2019 and enacted in January 2020. The proposition states that the law has two primary purposes. Firstly, it establishes the responsibilities of individual researchers and research institutions to ensure that research is conducted according to good scientific practices. Secondly, it regulates how accusations of research misconduct should be handled. The law expands on the Swedish *Higher Education Ordinance*^7^, which provided research institutions with the ability to investigate accusations of misconduct until the introduction of the new law.

#### Key Terms and Scope

The law seeks to ensure that research is conducted following *good scientific practice*, defined as “the moral practice that is developed when various actors in research, in dialogue with society, reflect critically about the conduct of research” (p. 14), and as “The sum of the ethical demands on how research should be conducted” (p. 32). Severe breaches of good scientific practices are labeled *misconduct*. The *Swedish National Board for Assessment of Research Misconduct* provides the following translation of the definition of misconduct found in the law:

A serious breach of good scientific practice in the form of fabrication, falsification or plagiarism that is committed intentionally or with gross negligence in the planning, performance or reporting of research^8^

In other words, the law defines misconduct as FFP. However, the proposition adds that plagiarism can sometimes be less severe and does not always qualify as misconduct, while fabrication and falsification should always be considered severe.

While misconduct is defined as FFP, these are not the only forms of deviance from good research practices recognized by the law. When it comes to the content of good research practices, the proposition to Parliament explicitly acknowledges that these are almost impossible to define. Firstly, these are formalized partially in “thousands of different policy documents. Some rules are shared by all research, while others are applicable to specific fields” (p. 44). They are thus not possible to summarize nationally. Secondly, good research practices develop over time. Therefore, “… it has to be the research community together with various authorities and legal entities that through practice decides what should count as good research practices” (p. 32). The proposition even recognizes that FFP is underdetermined. The definition is supposed to emerge through practice and the application of the law.

#### Responsibilities

The three primary actors discussed in the proposition are researchers, research institutions, and the *Swedish National Board for Assessment of Research Misconduct*. Additionally, the research community’s responsibility for developing good practices is mentioned briefly, and the Swedish administrative courts are given the role of handling appeals of the decisions of the national board. The law determines that individual researchers are responsible for following good research practices. Firstly, this involves attaining knowledge about relevant laws and codes of conduct and attending training provided by the research institutions where they work. Secondly, it is a matter of exerting one’s moral judgment as a researcher since researchers have a significant amount of freedom in their work. For a researcher, following the rules alone is therefore not enough to fulfill one’s responsibilities.

Swedish research institutions are also responsible for maintaining good research practices, but their responsibility is primarily to ensure that certain conditions are in place. Firstly, they are responsible for preventing breaches of good research practices. Here, the law mentions good supervision of PhD-students, education of staff, promoting discussions about research ethics, and proper documentation and archiving of research. These are just examples, and the proposition requires that research institutions engage actively in preventative work. Research institutions are also required to create systems for discovering breaches with good practices, including whistleblower protection, and they are required to put systems in place for handling breaches. If the breach falls within the law’s definition of misconduct, the research institution must report it to the national board for investigation, along with any relevant information. If the breach does not qualify as misconduct, the research institution must handle the case itself according to its routines. The proposition states that as such breaches come in many different forms, the rules for handling them are best set locally.

The national board is responsible for investigating accusations of research misconduct. Research institutions report these to the board, but the board can launch investigations on its own initiative if it receives anonymous tips or other forms of information that lead to suspicion. After the board’s investigation, the case is handed back to the research institution in question. The research institution must enact sanctions and inform affected parties such as journals and funders. Thus, the board only determines whether misconduct has taken place and does not punish perpetrators. To ensure that equal cases are given equal treatment, research institutions must report their follow-up of the board’s decision back to the board to maintain a national overview. The board has a secondary responsibility, which includes spreading awareness about good research practices, among other things, by writing an annual report about its work, analyzing its own decisions, and keeping research institutions informed.

## Discussion: Research Integrity and Ethics Regulation in Scandinavia

The new laws in Scandinavia adopt a similar approach in (1) the way they operationalize research integrity and ethics, (2) who they hold accountable, and (3) the tools and responsibilities they hand to the research community. All three take a hybrid approach to determining how research norms should be understood and enforced. On the one hand, they identify some of the most severe breaches of research norms as fabrication and falsification of data and plagiarism. These acts are deemed so grievous that they are covered explicitly by the laws, and all three countries have created national agencies with a mandate to investigate such cases. This move aligns with most codes of conduct and policies, as these tend to cover FFP and label these acts as misconduct (Horbach & Halffman, 2017; Resnik et al., 2015). The approach does not constitute an outright criminalization of misconduct, as it is not introduced in the penal codes of the three countries. However, it aims to ensure that FFP is identified and punished, and therefore comes close to criminalizing this form of misconduct.

On the other hand, the laws treat less severe breaches of integrity norms and ethics in a more open-ended way. The countries individually recognize the limited capacity of governments to regulate these directly under law. The propositions all include references to the changing nature of research, and conclude that the research community needs to develop and maintain its own norms. Less severe breaches are thus operationalized as what the research community presently holds to be unacceptable practices, which is a recognition that self-regulation is important in research.

The propositions each in their own way grant responsibilities and powers to research institutions for promoting integrity and ethics. While they all find that researchers must engage in self-regulation, they also mobilize the organizational level as they see self-regulation as insufficient by itself when it comes to ensuring integrity and ethics. This is an example of meta-regulation, in that they try to overcome the difficulties in regulating research by requiring actors with better insight into the relevant norms to take on the role as regulators.

In practice, the freedom of the research community to form and enforce its own norms is still subject to codification. The respective governments prefer for research institutions to formalize the norms in codes of conduct. However, this codification is supposed to be more sensitive to local conditions and practices than criminalization could be. According to the propositions, this approach allows the rules to be changed and updated rapidly, if needed, to allow for methodological innovations and the development of new norms. To ensure that research institutions can handle this responsibility, they are given the responsibility to establish their own capacity for investigating accusations, and they are required to establish guidelines for how accusations should be handled.

Giving much of the responsibility for promoting integrity and enforcing integrity norms to research institutions and the research community, thus attempting to deal with unwanted behavior through meta-regulation, has the potential to avoid some of the difficulties described in the background section of this paper. Under these conditions, researchers are held accountable if they do not keep up with the relevant norms. Therefore, the moral work they must engage in should not be replaced by a bureaucratic logic, which Heimer (2010) warns that more legalistic approaches have the potential to do, as they are required to maintain an active engagement with the norms of their field.

The laws both empower research institutions and formalize their role in promoting research integrity. These are empowered in the sense that the law gives the codes of conduct they develop legal backing. They also get a legal foundation for sanctioning researchers that break with recognized norms or good research practices, along with a right and a duty to promote a culture for ethics. As they are closer to the practice of research than policymakers, research institutions are better positioned to take both the continual development of research norms and the diversity of research norms between different disciplines into account when developing guidelines, handling accusations, or developing preventative measures.

While there is significant overlap between the three systems, there are some important differences. They vary in the terms they use. All explicitly condemn FFP, but where the Norwegian system is concerned with recognized research ethical norms, Sweden uses good research practices as the most central term. In Denmark, the focus is on questionable research practices, which is a negative approach, as the focus is on unwanted forms of behavior. Another difference is that the Norwegian law covers private institutions that have research as their primary purpose, while the Swedish law only covers publicly owned institutions.^9^ The Danish law covers private research that the government wholly or partially finances. A final relevant difference here is that the Norwegian and Swedish law covers both research ethics and the handling of what the literature tends to label as misconduct, while the Danish law tries to treat research ethics as a separate matter and focus mainly on misconduct. The Danish emphasis on compliance over ethics stems from the fact that their current approach builds on an earlier law. Their previous law implemented principles imported from the USA, which has a more legalistic and compliance-oriented approach (Vinther, 2016). This focus is expressed in the fact that the Danish proposition does not discuss the responsibilities of individual researchers, except by establishing that certain forms of research behaviors are unwanted. In contrast, both the Norwegian and Swedish propositions spend a significant amount of space discussing this topic.

### Trade-offs and Limitations

Some potential trade-offs and limitations are coming into the foreground now that the laws are put into practice. A recent report from the Science Ombud at the University of Oslo, an independent authority that researchers at that university can receive help from when it comes to integrity questions, states that “In the cases the Ombud has handled it is unclear which norms apply, who has recognized them, and how they can be applied for guidance and negotiation in specific cases”.^10^ Therefore, the formulation used in the new Norwegian legislation, “recognized research ethical norms,” is perhaps not sufficiently action guiding for all researchers. Researchers are a heterogenous group, and some researchers might be more comfortable working under clear guidelines. A similar issue has emerged in Denmark. Davies and Lindvig (2021) have shown that the interpretation of the Danish code of Conduct, which is formalized by the Danish law and forms the backbone of that system, varies from context to context and thus does not necessarily give clear guidance in practice.

It is worth noting that the American system thirty years ago included an open-ended formulation similar to the Scandinavian ones used to describe less serious examples of unwanted behavior. In 1989, the *United States Public Health Service* (PHS), a major funder of science, defined misconduct as FFP and “other practices that seriously deviate from those that are commonly accepted in the scientific community” (Cited in Resnik, 2003, p. 126). This definition is almost identical to the Norwegian one, and it is written in the same spirit as the Danish and the Swedish ones. According to Resnik (2003), such an open definition caused significant irritation among researchers and research institutions, as they found it too vague. Further American policies dropped it as a result. It remains to be seen whether the Scandinavian countries will be able to maintain this approach. The experience from Oslo and Denmark can be an early warning that the new laws run the risk of ending like the American one. However, by empowering institutions, through giving them tools and power to develop, promote, and enforce their norms, Scandinavian institutions are perhaps in a better position than the Americans when it comes to making sure that an open approach can work.

Another potential issue is that elevating FFP to law, as the three countries have done, can both be too inclusive and too exclusive at the same time. Bülow and Helgesson (2019) argue that other forms of misconduct can have just as severe consequences as that category. They use the example of withholding research results in biomedical fields, which can potentially lead to the loss of life. Due to its consequences, this is a much more severe issue than, for example, plagiarism in philosophy. The Norwegian law attempts to take this issue into account by including other forms of serious deviance from recognized research ethical norms when defining misconduct. In the proposition, such deviance is defined as severe when the act in question directly or indirectly impacts research results. The proposition is, unfortunately, confusing on this point, as it contradicts itself. It lists some examples of severe breaches, and the list includes acts like undue distribution of authorship and self-plagiarism, forms of misconduct that do not necessarily disrupt research results. This approach, therefore, makes things more unclear rather than solving the issue. The Swedish proposition has a more adaptive approach, as it states that the content of research norms will be determined through practice. This also goes for FFP, which they claim are underdetermined in the text of the law. Its content is supposed to be developed through the application of the law by the institutions and the courts.

Implementation is another important issue. In interviews conducted in Denmark, most of Davies’ (2019) informants told her that they were unaware of the Danish code of conduct. The informants who knew about the code expressed that they found it irrelevant to their work. This study is not representative, but it raises concerns that it is difficult to disseminate and get acceptance for the norms and codes that the Scandinavian countries promote, especially in Denmark, where they emphasize national guidelines that are some distance away from the practice of research. The Office of the Auditor General of Norway (2021) recently audited the implementation of the Norwegian law and gave it the lowest score possible. They found that research institutions had mostly failed to comply with the law, when it comes to ensuring that researchers receive training in research ethics and introducing adequate systems for the discovery and handling of misconduct. Why the implementation has not lived up to the intentions of the law so far should be the study of further research, but the audit shows that the research institutions are struggling to translate some of the law’s principles into practice.

Davies (2019) points to a more fundamental issue as well. It is not clear whether the Danish approach can solve the challenge of research misconduct without addressing its fundamental drivers, like competition and temporary contracts (Anderson et al., 2007). Such issues see some discussion in the propositions included in this paper. For example, in the Norwegian legislation, organizational factors must be evaluated when misconduct occurs, and research institutions are encouraged to be careful about introducing incentives that can promote unwanted behavior. However, this perspective is only discussed briefly. The Scandinavian legislation does not aim to deal with the fundamental drivers of misconduct and takes the current system of organizing and funding research for granted.

## Concluding Remarks

The Scandinavian countries have reformed their research integrity and ethics legislation in a way where they recognize the difficulty in formalizing such norms. Research is a diverse endeavor, with continuously developing norms, which are difficult to capture in law. While the three countries' governments believe that they have identified the most egregious forms of misconduct, FFP, and have condemned these forms of misconduct explicitly in their respective legislation, they approach other types of unwanted behavior with more modesty. Rather than establishing comprehensive lists of good practices and unwanted behavior, they recognize that it should be up to the research community itself to produce and enforce its own norms.

Adopting this approach does not mean that the respective governments are unconcerned with other questionable or unethical research practices. Their response is to give researchers and research institutions formal obligations to familiarize themselves with the proper norms and follow them. They provide a set of tools to research institutions to help them live up to this responsibility, such as establishing national investigatory bodies and giving research institutions responsibilities for establishing routines for investigation of accusations of unwanted research behavior. This approach balances the need for academic freedom and the dynamic nature of research norms with the need to control misconduct and questionable research practices, through a form of meta-regulation. The approach has some potential trade-offs and limitations. It is potentially not action-guiding enough. Furthermore, elevating FFP as the most egregious forms of misconduct can be arbitrary, as other forms of fraud or dishonesty can be just as severe in their consequences. Finally, it is not clear that the new legislation sufficiently addresses the fundamental issues that produce misconduct.

## Data Availability

Not applicable.

## Footnotes

1 *Stakeholders Acting Together On the ethical impact assessment of Research and Innovation.*

2 *Promoting Integrity as an Integral Dimension of Excellence in Research*. I conducted my doctoral work as a member of the PRINTEGER-project, but the present paper was produced independently of the consortium.

3 Representatives from Finland and Iceland participated, along with representatives from the three countries discussed in this paper.

4 No translation of the law or its name exist. The Danish version can be found here https://www.retsinformation.dk/eli/lta/2003/405.

5 The translation of both definitions was copied from the website of the Danish Committee on Research Misconduct https://ufm.dk/en/research-and-innovation/councils-and-commissions/The-Danish-Committee-on-Research-Misconduct?set_language=en&cl=en.

6 An unofficial translation of the statutes, which I use in this section, can be found here: https://www.uhr.se/en/start/laws-and-regulations/Laws-and-regulations/act-on-responsibility-for-good-research-practice/..

7 An unofficial translation of the document can be found here https://www.uhr.se/en/start/laws-and-regulations/Laws-and-regulations/The-Higher-Education-Ordinance/..

8 https://oredlighetsprovning.se/beslut..

9 Sweden is currently considering whether private institutions should be regulated as well.

10 https://www.uio.no/om/organisasjon/vitenskapsombud/arsrapport/arsrapport-2019-fra-vo-til-universitetsstyret.pdf—my translation.

## References

[CR1] Anderson MS, Ronning EA, De Vries R, Martinson BC (2007). The perverse effects of competition on scientists’ work and relationships. Science and Engineering Ethics.

[CR2] Asdal, K., & Reinertsen, H. (2021). *Doing document analysis: A practice-oriented method*. Sage

[CR3] Baldwin M (2018). Scientific autonomy, public accountability, and the rise of ‘Peer Review’ in the cold war United States. Isis.

[CR4] Beauchamp TL, Childress JF (2009). Principles of biomedical ethics.

[CR5] Bülow W, Helgesson G (2019). Criminalization of scientific misconduct. Medicine, Health Care aNd Philosophy.

[CR6] Coglianese, C., & Mendelson, E. (2010). Meta-regulation and self-regulation. In: M. Cave, R. Baldwin, & M. Lodge (Eds.), *The Oxford handbook on regulation*. Oxford University Press

[CR7] Creswell, J. W. (2014). *Research design—Qualitative, quantitative and mixed methods approaches*. 4th edn. SAGE 10.7748/nr.12.1.82.s2 28718745

[CR8] Dal-Ré R, Bouter LM, Cuijpers P, Gluud C, Holm S (2020). Should research misconduct be criminalized?. Research Ethics.

[CR9] Davies SR (2019). An ethics of the system: Talking to scientists about research integrity. Science and Engineering Ethics.

[CR10] Davies SR, Lindvig K (2021). Assembling research integrity: Negotiating a policy object in scientific governance. Critical Policy Studies.

[CR45] Dorbeck-Jung Bärbel, Shelley-Egan Clare (2013). Meta-Regulation and Nanotechnologies: The Challenge of Responsibilisation Within the European Commission’s Code of Conduct for Responsible Nanosciences and Nanotechnologies Research. NanoEthics.

[CR11] Fanelli D (2009). How many scientists fabricate and falsify research? A systematic review and meta-analysis of survey data. Edited by Tom Tregenza.. PLoS ONE.

[CR12] Freckelton IR (2016). Scholarly misconduct: law, regulation, and practice.

[CR13] Fuster, G. G., & Gutwirth, S. (2016). Legal analysis (Deliverable 2.4). PRINTEGER. https://printeger.eu/wp-content/uploads/2017/02/D2.4.pdf

[CR14] Gilad S (2010). It runs in the family: Meta-regulation and its siblings. Regulation and Governance.

[CR15] Heimer CA (2010). The unstable alliance of law and morality.

[CR16] Hiller KM, Haukoos JS, Heard K, Tashkin JS, Paradis NA (2005). Impact of the final rule on the rate of clinical cardiac arrest research in the United States. Academic Emergency Medicine.

[CR18] Horbach SPJM, Halffman W (2017). Promoting virtue or punishing fraud: Mapping contrasts in the language of ‘scientific integrity’. Science and Engineering Ethics.

[CR19] Israel, M. (2015). *Research ethics and integrity for social scientists: beyond regulatory compliance*. Sage.

[CR20] Klitzman R (2011). Views and experiences of IRBs concerning research integrity. The Journal of Law, Medicine & Ethics.

[CR21] Kuroki T (2018). New classification of research misconduct from the viewpoint of truth, trust, and risk. Accountability in Research.

[CR22] Lamont, M. (2009). *How professors think: Inside the curious world of academic judgement*. Harvard University Press.

[CR23] Li D, Cornelis G (2020). Differing perceptions concerning research misconduct between China and flanders: a qualitative study. Accountability in Research.

[CR24] *Lovforslag Nr. L 117 (2016–2017)—Lov Om Videnskabelig Uredelighed m.v.* https://www.ft.dk/samling/20161/lovforslag/L117/som_fremsat.htm.

[CR25] Macfarlane B (2009). Researching with integrity: The ethics of academic enquiry.

[CR26] Merton, R. K. (1973). The normative structure of science. In Norman W. Storer (Ed.), *The sociology of science—theoretical and empirical investigations* (pp. 267–278). The University of Chicago Press.

[CR28] Ministry of Higher Education and Science. (2014). Danish code of conduct for research integrity. https://ufm.dk/en/publications/2014/the-danish-code-of-conduct-for-research-integrity.

[CR29] NordForsk. (2015). Research integrity in the Nordic Countries—National systems and procedures. https://old.nordforsk.org/no/publikasjoner/publications_container/research-integrity-in-the-nordic-countries-2013-national-systems-and-procedures.

[CR30] Office of the Auditor General of Norway. (2021). *Riksrevisjonens rapport om forskningsetikk i universitets- og høyskolesektoren*. https://www.riksrevisjonen.no/rapporter-mappe/no-2021-2022/undersokelse-av-forskningsetikk-i-universitets-og-hoyskolesektoren/

[CR31] Peels R, de Ridder J, Haven T, Bouter L (2019). Value pluralism in research integrity. Research Integrity and Peer Review.

[CR32] *Prop 158 L (2015–2016) - Lov om organisering av forskningsetisk arbeid (forskningsetikkloven)*. https://www.regjeringen.no/no/dokumenter/prop.-158-l-20152016/id2511345/.

[CR33] *Prop. 2018/19:58 - Ny ordning för att främja god sed och gantera oredlighet i forskning*. https://www.regeringen.se/rattsliga-dokument/proposition/2019/03/prop.-20181958/.

[CR34] Rangi, S., & Warso, Z. (2015). A report on the legal frameworks that guide or constrain ethical procedures within research within the EU (Deliverable 3.1). SATORI. https://satoriproject.eu/media/SATORI-Deliverable-3.1-.pdf.

[CR35] Redman BK, Caplan A (2021). Should the regulation of research misconduct be integrated with the ethics framework promulgated in *The Belmont Report*?. Ethics and Human Research.

[CR36] Resnik DB (2003). From Baltimore to bell labs: Reflections on two decades of debate about scientific misconduct. Accountability in Research.

[CR37] Resnik DB, Rasmussen LM, Kissling GE (2015). An international study of research misconduct policies. Accountability in Research.

[CR38] Schrag ZM (2009). How talking became human subjects research: The federal regulation of the social sciences, 1965–1991. Journal of Policy History.

[CR39] Schrag ZM (2010). Ethical imperialism: Institutional review boards and the social sciences, 1965–2009.

[CR40] Shaw D (2018). The quest for clarity in research integrity: A conceptual schema. Science and Engineering Ethics.

[CR41] Steneck NH (2006). Fostering integrity in research: Definitions, current knowledge, and future directions. Science and Engineering Ethics.

[CR42] Stolberg HO, Norman G, Trop I (2004). Randomized controlled trials. American Journal of Roentgenology.

[CR43] Vinther, T. (2016). Arbeidet med vitenskapelig uredelighet i et komparativt lys. In *Vitenskapelig (u)redelighet*. Cappelen Damm Akademiske. https://www.cappelendammundervisning.no/_vitenskapelig-uredelighet-9788202522063.

[CR44] World Medical Association (2013). World Medical Association Declaration of Helsinki ethical principles for medical research involving human subjects. JAMA Journal of the American Medical Association.

